# Personality cannot be predicted from the power of resting state EEG

**DOI:** 10.3389/fnhum.2015.00063

**Published:** 2015-02-13

**Authors:** Kristjan Korjus, Andero Uusberg, Helen Uusberg, Nele Kuldkepp, Kairi Kreegipuu, Jüri Allik, Raul Vicente, Jaan Aru

**Affiliations:** ^1^Institute of Computer Science, University of TartuTartu, Estonia; ^2^Institute of Psychology, University of TartuTartu, Estonia; ^3^Doctoral School of Behavioral, Social and Health Sciences, University of TartuTartu, Estonia; ^4^Estonian Academy of SciencesTallinn, Estonia; ^5^Institute of Public Law, University of TartuTartu, Estonia

**Keywords:** personality, EEG, resting state, machine learning, prediction

## Abstract

In the present study we asked whether it is possible to decode personality traits from resting state EEG data. EEG was recorded from a large sample of subjects (*n* = 289) who had answered questionnaires measuring personality trait scores of the five dimensions as well as the 10 subordinate aspects of the Big Five. Machine learning algorithms were used to build a classifier to predict each personality trait from power spectra of the resting state EEG data. The results indicate that the five dimensions as well as their subordinate aspects could not be predicted from the resting state EEG data. Finally, to demonstrate that this result is not due to systematic algorithmic or implementation mistakes the same methods were used to successfully classify whether the subject had eyes open or closed. These results indicate that the extraction of personality traits from the power spectra of resting state EEG is extremely noisy, if possible at all.

## INTRODUCTION

Personality can be defined as a relatively stable pattern on thinking, feeling, and acting. These patterns can be explained by the idea of personality traits – underlying mechanisms that cause variation in observable personality characteristics (Deary, 2009). According to a dominant five factor model (FFM), observable personality is mostly determined by five major traits – Neuroticism, Extraversion, Openness, Agreeableness, and Conscientiousness (McCrae and John, 1992; McCrae and Costa, 2008). Their relatively high cultural universality, temporal stability, and heritability suggest that the Big Five traits may represent some sufficiently stable parameters of fairly specific brain networks (Corr, 2004; DeYoung and Gray, 2009; Kennis et al., 2012).

If traits indeed reflect individual differences in tonic brain function, then measures of baseline brain activity may provide a direct way for personality assessment. A relatively cost-effective way for quantifying the biological origins of traits could thus be developed by finding reliable correlates of trait levels from the resting state EEG signal. However, existing attempts to do this have generally yielded mixed results. For instance, an early hypothesis relating Extraversion to baseline brain arousal turned out to be a gross over-simplification (Stelmack, 1990). Another influential idea linking anterior asymmetry in EEG alpha (8–12 Hz) band power to individual differences in approach and avoidance systems of the brain (Davidson, 2001; Coan and Allen, 2002), has similarly not been confirmed using meta-analytic methods (Wacker et al., 2010).

On the other hand, new findings keep suggesting novel candidate parameters of resting state EEG as potential correlates of certain personality traits. For instance, mid-frontal theta power has been found to co-vary with Extraversion (Knyazev, 2009; Wacker et al., 2010). Meanwhile, the extent of negative relationships between power in lower (delta and theta) and higher (alpha and beta) frequency bands (i.e., cross-frequency anti-coupling) seems also to index approach-avoidance related individual differences (Schutter and Knyazev, 2012). Then again, this hopeful state of affairs might simply reflect the fact that the proposed correlates are still novel and negative findings remain to be published.

Most of the existing research on resting state EEG correlates of personality traits has been conducted in a hypothesis-driven way, concentrating usually on a single parameter at a time. An alternative approach would be to use data-driven techniques to first of all assess the extent to which resting state EEG signal contains information on personality and then search for relevant correlates in a more comprehensive and systematic manner. The main aim of the present study is to test such an approach. To that end we used classifiers, mathematical models that map input data to a set of classes or labels, to predict personality traits from resting state EEG signals. The classifiers were first trained using a set of data with known classes and then their performance was evaluated on data not used for the training phase.

A secondary aim of the present study is to investigate which level of personality trait description is best suited for relating to resting state EEG. In an influential paper, DeYoung et al. (2007) identified two lower-order aspects for each of the Big Five traits. Subsequent research has demonstrated that this level of trait descriptions may have more homogenous brain origins (e.g., DeYoung, 2013). We therefore test if the information contained within resting state EEG is more reliably related to the 10 aspects compared to the five traits of the Big Five.

## RESULTS

We analyzed a large dataset collected from 289 participants. This dataset consisted of eyes open and eyes closed resting state EEG recordings (32 active electrodes) together with Big Five personality scores assessed using a validated self-report questionnaire.

In the first part of the analysis, we trained statistical classifiers to map the features of the resting state EEG to personality scores of individual subjects. In particular, we used the power spectra of the EEG signals as the basis for the features or explanatory variables. The predicted variable consisted of the binarization (using a median split) of the score for each personality trait (see Materials and Methods, *Personality measures*). Given the exploratory nature of the analysis we scanned different combinations of classifier parameters and features from the EEG power spectra to find the configuration that best classified each personality trait. To avoid *cherry-picking* or over-fitting the results we always assessed the selected classifiers on a separate subset of subjects.

Thus, we used a nested cross-validation approach, which has inner and outer loops of cross validation. Inner 10-fold cross-validation loop is used to choose the hyper-parameters of the classifier (including different data pre-processing options, dimensionality reduction, and the choice between linear and non-linear support-vector-machine (SVM) classifier, see Materials and Methods). The classifier that performed the best (by minimizing misclassification rate) for each personality trait is used for the outer loop to estimate the misclassification rate of the selected classifier. So, the best hyper-parameters are chosen 10 times and the final misclassification rates represent the averages over these 10 partitions (see Materials and Methods, Classification of the Personality Traits).

**FIGURE 1:**
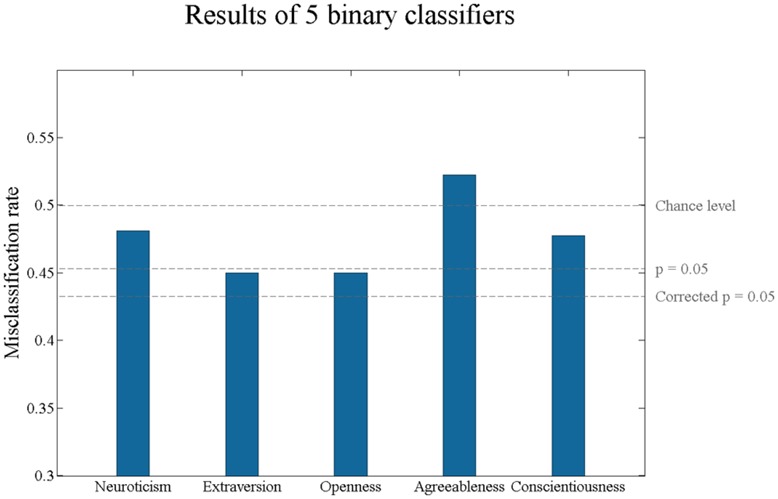
**Misclassification rates of Big Five personality traits.** The personality scores have been binarized with a median-split. None of the misclassification rates of five personality traits is statistically significant after Bonferroni or false discovery rate correction at 0.05.

The results for the binary classification of the test subjects are shown on **Figure 1**. Although the best classification rates observed for Extraversion and Openness initially reached significance, they did not remain significant after Bonferroni or after false discovery rate correction (binomial test, corrected *p* > 0.05). This indicates that none of the personality traits were correctly classified from any of the explored combinations of resting state EEG features.

Although the binarization of the personality scores is conceptually simple, robust, and easy to interpret, it also leads to a loss of information that might hamper the predictive power of the classifiers. Thus, we next tried to predict the personality score of each trait as a continuous variable. For this case, instead of SVM classifiers, we used LASSO and elastic nets models of regressions. These models of regression include a penalty term that reduces the number of explanatory variables used while minimizing the error of the regression. Given the large number of features considered in our analysis, using sparse regression models allows us to control for over-fitting errors, as sparse models often tend to generalize better for novel data. Following the same nested cross-validation approach as in the binary classifier pipeline, the results showed that none of the mean squared errors was significantly better (all *p* > 0.4) than the null model hypothesis that the best prediction is the mean of the personality scores. This result indicates that essentially no additional information could be predicted from the EEG power spectrum features by using this approach.

Arguably, some of variance in raw personality trait scores is induced by age and gender differences between participants that may have little to do with underlying personality differences. We therefore tested if removing such variance would improve the information yield of resting state EEG. All trait scores were normalized in relation to age- and gender-specific means and standard deviations (see Methods, *Personality Measures*). The repetition of the binary classification analysis with these normalized scores still provided misclassification errors that were not statistically significant (all *p* > 0.1).

**FIGURE 2:**
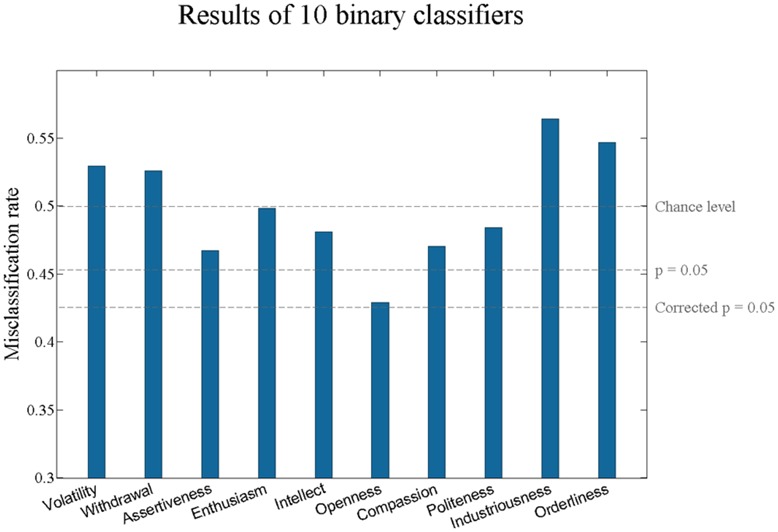
**Misclassification rates of lower-order aspects of Big Five personality traits.** The aspect scores have been binarized with a median-split. Although the *p*-value of the misclassification rate for *Openness* is below 0.05, it does not remain significant after Bonferroni or false discovery rate correction.

Given the possibly enhanced homogeneity of the brain origins of lower-level aspects of personality traits, we next attempted to predict the scores for the 10 lower-order aspects of the Big Five. We again used the binary classifier with the similar pipeline as for the five superordinate traits. The results are shown in **Figure 2**. As can be seen from **Figure 2**, one aspect, *Openness,* is statistically significant at the uncorrected level. However, after Bonferroni or after false discovery rate correction none of the *p* values remain significant. Therefore, we could not classify the lower-order aspects of Big Five personality traits from the power spectra of resting state EEG.

To test the general validity of our analysis pipeline, we applied it to a situation where clear classifiable information was present in the dataset. To that end we tried to classify whether the eyes of the subject were open or closed.

The subjects were assigned into two classes: for half of the subjects eyes open data were used and for another half eyes closed data were used. Using the same pipeline with nested cross-validation of 324 models (half of original size because only one type of data is available this time) on 289 subjects, the achieved classification rate of the classifier was 85.8% (misclassification rate of 14.2%, *p* ≪ 0.001). This result indicates that when a clear pattern of information was present in the data, our algorithm was able to extract it and perform almost optimally. This result strongly indicates that the failure to predict personality traits from spectral components of resting state EEG is probably not due to systematic algorithmic or implementation mistakes.

## DISCUSSION

In the present study we asked whether it is possible to predict the Big Five personality traits from resting state EEG data. Previous studies investigating the neurobiological correlates of personality have focused on single parameters such as the power of specific frequency components. Here we approached the research question from the data-driven perspective, using machine learning to assess how much information about personality traits is contained in the spectral dynamics of the resting state EEG. In particular, we analyzed the data from a wide power spectrum and all the 32 electrodes. We used nested cross-validation on 289 subjects to estimate the accuracy of the classifiers.

Our results indicated that it is not possible to predict the Big Five personality traits from the power spectra of resting state EEG data. We furthermore showed that our classifiers are also not successful in predicting the 10 lower-order aspects of Big Five. Although for trait Extraversion and for trait as well as aspect *Openness* the uncorrected *p*-value of classification accuracy was lower than 0.05, these results did not survive a correction for multiple comparisons. We confirmed that predictions could not be enhanced by analyzing personality traits as continuous variables or removing age and gender-related variability. Furthermore, for a previous version of this manuscript we analyzed the data with different preprocessing parameters, but nevertheless observed the same pattern of negative results.

In general, although our sample size is large in the context of EEG measures, machine learning techniques are typically applied to larger data sets. In addition, any machine learning approach might suffer from several trade-offs and arbitrariness at each of its different stages (data preprocessing, feature extraction, feature selection, model selection, model training, validation). Nevertheless, the pipeline devised for the current study was able to discover meaningful signals in the available amount of data. More specifically, using the same methods, we were able to differentiate eyes open recordings from eyes closed ones with a misclassification rate of 14%. This finding strongly suggests that the inability to classify the personality traits was not caused by our analysis pipeline.

Given the comprehensive statistical approach used in this study, the results, despite being negative, have implications for personality neuroscience. On the one hand, the limitations of the recording, and analysis techniques used here can be informative. Our study relied on the power spectrum of each channel as the basis for selecting features or explanatory variables that may predict personality traits. Spectral rather than temporal characteristics of resting state EEG were explored as all points in time are statistically equivalent in this type of signal (as opposed to evoked or induced potentials). Nevertheless, it is possible that features not captured by power such as oscillatory phase or temporal correlations in channel or source space might add some extra information. For instance, some studies have found relationships between trait measures such as Neuroticism and negative correlations between amplitudes of higher (alpha/beta) and lower (theta/delta) frequencies (Schutter and Knyazev, 2012). Note however that in many of these studies, power levels in certain frequencies are also related to the cross-frequency coupling indices as well as to the relevant trait measures (e.g., Knyazev et al., 2003). It thus remains to be seen if different feature extraction strategies would increase the success of classifying personality scores from resting state EEG.

Another technical explanation for the current findings might be the limited nature of EEG as a measure of brain processes. EEG is sensitive to only a subset of electrical events in the brain, probably reflecting the synchronized local field potentials of suitably aligned cortical pyramidal neurons (Lopes da Silva, 2013). There is some evidence suggesting that the trait-relevant differences may instead be found on the level of brain structure (e.g., DeYoung et al., 2010) or activation in subcortical areas that are unlikely to contribute directly to scalp EEG (e.g., Cunningham et al., 2010).

These methodological considerations notwithstanding, the present findings may also have conceptual implications. Individual differences in brain processes can either be stable dispositions evident in majority of situations (i.e., situation-independent traits) or characteristic responses to specific stimuli (situation-dependent traits; Mischel and Shoda, 1998; Fleeson and Noftle, 2009; Stemmler and Wacker, 2010). Given the lack of stimulation, the resting state measurement is optimized for discovering the former rather than the latter type of traits. In this framework, the failure to relate resting state EEG to Big Five personality traits documented here suggests that the brain substrate of personality might involve situation-dependent responsiveness rather than differences in baseline activity.

A relevant example can be found from the literature relating anterior EEG asymmetry to personality. Although many researchers implicitly assume the resting condition to be optimal for quantifying trait asymmetry, there is evidence that trait-related changes in asymmetries are best captured in response to some relevant stimulation (Coan et al., 2006). Furthermore, the correlations between traits and resting asymmetry that do emerge may also be driven by the situational features of the resting measurement occasion. For instance, anterior asymmetry responses distinguished male participants based on their trait Defensiveness only at the presence of an attractive female experimenter (e.g., Kline et al., 2002). Given that we analyzed data from different studies collected by different experimenters, such situational factors should have fairly randomly distributed effects within the present data. This reduces the risk of situation-mediated EEG-personality covariance being registered as evidence for correlations on the trait level.

In summary, regarding the discovery of neural bases of personality traits, the present null-finding may constitute a false negative in the sense that technical limitations of EEG recording and/or the employed analysis techniques precluded us from detecting true trait differences in brain activity. In addition, even while the size of the current sample (289 participants) is similar to some normative datasets, we cannot exclude the possibility that more data would have provided significant results. On the other hand however, the results might imply that the brain substrate of personality may exist on the level of characteristic responsiveness rather than baseline activity, in line with several findings in modern personality neuroscience (Stemmler and Wacker, 2010).

## MATERIALS AND METHODS

### SAMPLE AND PROCEDURE

The sample consists of 289 participants of 12 different cognitive EEG experiments conducted at the University of Tartu (102 males; age range 18–42, *M* = 22.0, σ = 3.6). All participants were volunteers recruited among university students and the general population. All measurements were approved by the Ethics Review Board of Tartu University. Only subjects who completed a personality questionnaire and had at least 50% of the originally recorded EEG data retained after artifact rejection were included in the sample.

The resting state EEG data analyzed here were collected prior to all other experimental tasks. Two different measurement protocols were used. In five experiments the resting state signal was recorded in two contiguous sections – one with eyes open and the other with eyes closed. Each section lasted either for 1 min (in one experiment with 75 participants) or 2 min (in four experiments with 85 participants). In the remaining seven experiments (129 participants) three separate 1 min measurements with eyes open and eyes closed were interleaved resulting in 3 min in total for both the eyes open and eyes closed conditions.

The measurements took place in a dimly lit and quiet room. Participants sat in a comfortable office chair 1 or 1.15 m away from a computer screen. They were instructed to relax and avoid excessive body and eye movements. During the eyes open condition they were also required to fixate on a black cross in the middle of a gray screen. After instructions, participants remained alone in the room during the actual recording.

### EEG RECORDING AND PREPROCESSING

A BioSemi ActiveTwo (BioSemi, Amsterdam, Netherlands) active electrode system was used to record signals from 32 scalp locations, two reference electrodes placed on earlobes and four ocular electrodes (above and below the left eye and near the outer canthi of both eyes). The data were recorded with 0.16–100 Hz band-pass filter and 1024 or 512 Hz sampling rate.

Oﬄine pre-processing was implemented in Matlab (MathWorks, USA) and EEGLAB (Delorme and Makeig, 2004) software. The data were re-referenced to digitally linked earlobes and resampled to 512 Hz as necessary. Eye-movement artifacts were corrected using independent component analysis (ICA). Infomax ICA algorithm was trained for each subject on a separate copy of data that was first high-pass filtered (half-amplitude cut-off at 1 Hz) and then cleaned of large artifacts by screening 1 s epochs for excessive muscular noise (EEGLAB rejspec 15–30 Hz; ±45 dB). If more than 2% of epochs were marked for rejection based on a single channel, the channel was removed before rejecting the remaining epochs with artifacts. Independent components capturing eye-blinks as well as vertical and horizontal eye-movements were visually identified and removed before reconstructing the whole duration of unfiltered and unsegmented data (Debener et al., 2010). The ICA-pruned continuous data were cut into 2 s epochs and the mean voltage of each epoch was removed as a baseline. All segments where voltage fluctuations from the mean exceeded 100 μV in either direction were marked as artifacts. If this criterion was violated in only a single channel for more than 2% or the trials, this channel was removed before removing the remaining segments with artifacts. All rejected channels were spherically interpolated for ease of subsequent analyses (EEGLAB *eeg_interp*).

The power spectral density values were computed using the Fourier transform with Hamming tapered 2 s windows. Within all consecutively recorded stretches of data at least 3 s in duration, 50% overlapping windowing was used. The power estimates between 1 and 95 Hz were added and divided by the number of windows separately within eyes closed and eyes open condition.

### PERSONALITY MEASURES

**FIGURE 3:**
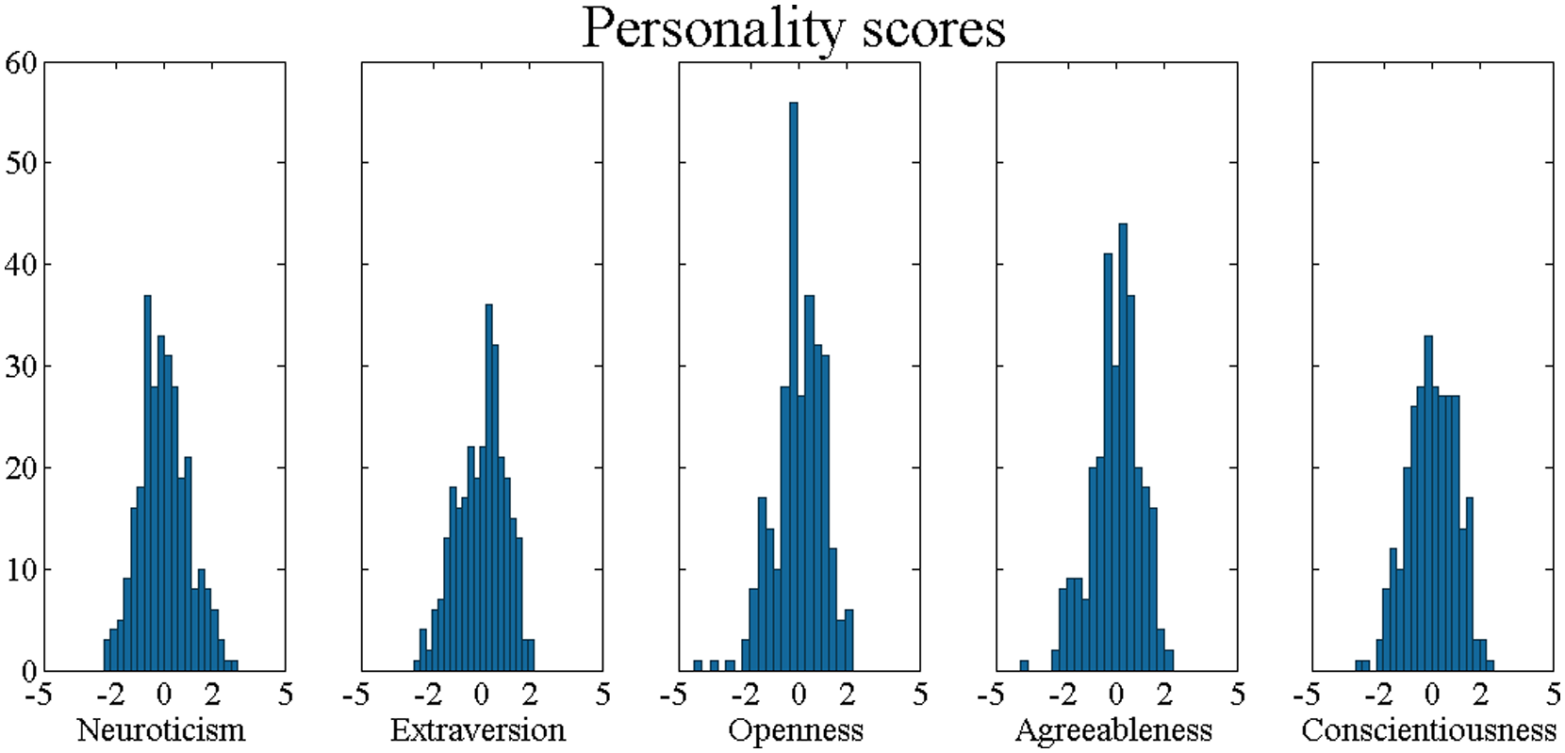
**Histograms of all five personality dimension scores.** Note that the distributions are rather normal and personality scores are normalized by subtracting the mean and dividing by the SD.

Prior to visiting the lab, participants filled in a personality questionnaire in a dedicated online environment (kaemus.psych.ut.ee). All 289 participants completed the EE.PIP-NEO inventory which uses 240 items to assess the five dimensions (see **Figure 3**) as well as 30 facets of the five factor model (FFM; Mõttus et al., 2006).

Scores for the 10 lower-order aspects of the Big Five were computed as averages of the relevant facet scores of the FFM (Judge et al., 2013):

•Volatility = N2, N5
•Withdrawal = N1, N3, N4, N6
•Enthusiasm = E1, E2, E5, E6
•Assertiveness = E3, E4, E5
•Intellect = O5
•Openness = O1, O2, O3, O4, O6
•Compassion = A1, A3, A6
•Politeness = A2, A4, A5
•Industriousness = C1, C4, C5
•Orderliness = C2, C3, C6

Median split was performed on each personality trait, resulting in two equal size classes “high” and “low” score for each personality trait.

### CLASSIFICATION OF THE PERSONALITY TRAITS

We used a nested cross-validation approach. For predicting an out of sample subject we used 10-fold cross-validation. In order to choose the best hyper-parameters we used again 10-fold cross-validation for 90% of the data. After finding the best combination of hyper-parameters we re-trained it on the same 90% of the data. Each of the five personality traits was treated separately. Matlab code for the nested cross-validation can be found from GitHub repository: https://github.com/kristjankorjus/PredictingPersonalityFromEEG

#### Nested cross-validation

In order to find the best hyper-parameters 10-fold cross-validation was used on the 90% of the data inside of cross-validation. The full list of hyper-parameters is specified here:

•different data types: using power spectra of eyes open data or eyes closed data (*two options*)
•pooling of electrodes: using all electrodes separately or taking regions of interests [left frontal (F3, F7, AF3, Fp1); right frontal (F4, F8, AF4, Fp2); mid-frontal (Fz, Cz, FC1, FC2); left central (FC5, CP5, T7, P7, C3); right central (FC6, CP6, T8, P8, C4); mid-parietal (Pz, CP1, CP2, P3, P4); and occipital (PO3, PO4, O1, Oz, O2)] (*two options*)
•pooling of frequencies: using all frequencies, using customized pooling for frequencies (taking information from spectrum averaged over all subjects and channels such that from 1 to 25 Hz bands of 0.5 Hz were used; from 25.5 to 35 Hz bands of 1 Hz; from 35.5 to 95 Hz bands of 10 Hz were used) or using 10 bands based on the literature (1, 1.5 to 4, 4.5 to 8, 8.5 to 12, 12.5 to 20, 20.5 to 30, 30.5 to 40, 40.5 to 50, 50.5 to 69.5, 70 to 95, all values in Hz; *three options*)
•normalization of the data: using the data without normalization, normalizing each row (each subject) by taking the *z*-score (subtracting the mean, dividing by standard deviation) or normalizing each column (each feature; *three options*)
•for reduction of dimensionality of the data, principal component analysis was used with different amount of total variance explained by principal components: 90% or 70% of variance explained. Or no principal component analysis was used at all (*three options*)
•value of the box constraint C for the soft margin of SVM: 0.01 or 100 (*two options*)
•type of kernel for SVM: no kernel (linear SVM) or a radial basis function (RBF) kernel (non-linear SVM) were used. In addition, the sigma parameter controlling the width of the RBF was selected among two possible values using randomized data as explained in the next paragraph (*three options in total*).

To find a suitable sigma parameter of RBF for our dataset we estimated how the over-fitting error for a known case depended on this parameter. In particular, we first randomized the classes of the dataset, and let the classifier train and predict the full data. For small values of the sigma, the classification error will tend to 0 indicating over-fitting – every neighborhood around a sample is classified with the label of the contained sample, and therefore lacking of any generalization power. As the sigma increased, the error rate started to increase. Sigma was fixed when the error rate reached 0.1 or 0.3 as these were optimistic prior beliefs about the final classification error.

In total, these 648 combinations of classifier hyper-parameters were explored for each personality trait. For the whole cross-validation phase, partitioning of data was fixed to make different hyper-parameters more comparable. The smallest misclassification error for each personality trait determined the set of hyper-parameters, which were used in testing.

#### Cross-validation

In order to estimate the misclassification error rate, each personality trait of 289 subjects was predicted with the best hyper-parameters found and model trained in the nested cross-validation phase.

Statistical significance was estimated using binomial test with the null hypothesis that two categories are equally likely to occur.

### ANALYZING CONTINUOUS DATA

Continuous scores were first normalized (mean was subtracted and divided by standard deviation). Instead of a SVM classifier, LASSO and elastic net regressions were used (Matlab function *lasso*). All hyper-parameters which were not related to the specifics of SVM classifiers, were scanned from the same range as described in the Cross-Validation section above. In addition, the *alpha* parameter was scanned with three options: 1, 0.5, and 0.01. Parameter *alpha* = 1 represents LASSO regression, *alpha* close to 0 approaches ridge regression, and the 0.5 represents elastic net optimization. For the regularization parameter, we used the recommended *lambda* such that MSE is within one standard error of the minimum (see *Lambda1SE* in the *lasso* documentation in MATLAB).

In the continuous case, the null hypothesis was that the best prediction for each personality trait, which minimizes the mean squared error, is the mean of the personality score. Statistical significance analysis was performed using a permutation test: scores were sampled with replacements from the score distribution.

### TESTING THE INFLUENCE OF AGE AND GENDER RELATED VARIABILITY

To test possible age and gender related systematic personality variability, all trait scores were normalized in relation to age- and gender-specific means and SD $\left(S_{norm}=\frac{S_{raw}-\mu_{reference}}{\sigma_{reference}}\cdot 10+50\right)$. The reference data were obtained from a normative sample of the EE.PIP-NEO (*n* = 1564; 889 males; age range 16–86; *M* = 15.8, σ = 12.1). Based on the age and gender of the participant, one of 20 reference groups were selected (9 age brackets between 15 and 59 with 5 year steps and one bracket for 60 and above). For 53 participants of the present sample for whom age data were unavailable, the sample mean age was used for reference group identification. Again, median split, and original pipeline described above was used.

### ASSESSMENT OF 10 LOWER-ORDER ASPECTS

Instead of five personality traits, each trait has a natural subdivision into two:

•neuroticism: *volatility* and *withdrawal*
•extroversion: *assertiveness* and *enthusiasm*
•openness to experience: *intellect* and *openness*
•agreeableness: *compassion* and *politeness*
•conscientiousness: *industriousness* and *orderliness*

All of the 10 sub-traits were classified using the original above-described pipeline.

### CLASSIFICATION OF EYES CLOSED VS EYES OPENED DATA

To test the validity of the whole pipeline, classification was performed in a situation with clear pattern of information present. In particular, the data were divided into two sets such that half of the subjects had eyes open data and another half had eyes closed, in total two classes. Notice that no subject appears in two classes. Thus we still have 289 subjects and two classes but for each subject the data is taken from either eyes open or eyes closed condition. Again, original pipeline was used.

## AUTHOR CONTRIBUTIONS

KK, AU, RV, and JA analyzed the data. AU, HU, NK, KK, and JA, organized the data collection. KK, AU, RV, and JA wrote the manuscript with input from all other authors.

## Conflict of Interest Statement

The authors declare that the research was conducted in the absence of any commercial or financial relationships that could be construed as a potential conflict of interest.
